# Deep learned tissue “fingerprints” classify breast cancers by ER/PR/Her2 status from H&E images

**DOI:** 10.1038/s41598-020-64156-4

**Published:** 2020-04-29

**Authors:** Rishi R. Rawat, Itzel Ortega, Preeyam Roy, Fei Sha, Darryl Shibata, Daniel Ruderman, David B. Agus

**Affiliations:** 1 Lawrence J. Ellison Institute for Transformative Medicine, University of Southern California, 12414 Exposition Blvd, Los Angeles, CA 90064 USA; 2 DASH Center at USC, 1002 Childs Way, MCB 114, Los Angeles, CA 90089-0005 USA; 3 Department of Pathology, University of Southern California Health Sciences Campus, NOR 1441 Eastlake Ave, Los Angeles, 90033 USA

**Keywords:** Cancer imaging, Breast cancer, Image processing, Machine learning

## Abstract

Because histologic types are subjective and difficult to reproduce between pathologists, tissue morphology often takes a back seat to molecular testing for the selection of breast cancer treatments. This work explores whether a deep-learning algorithm can learn objective histologic H&E features that predict the clinical subtypes of breast cancer, as assessed by immunostaining for estrogen, progesterone, and Her2 receptors (ER/PR/Her2). Translating deep learning to this and related problems in histopathology presents a challenge due to the lack of large, well-annotated data sets, which are typically required for the algorithms to learn statistically significant discriminatory patterns. To overcome this limitation, we introduce the concept of “tissue fingerprints,” which leverages large, unannotated datasets in a label-free manner to learn H&E features that can distinguish one patient from another. The hypothesis is that training the algorithm to learn the morphological differences between patients will implicitly teach it about the biologic variation between them. Following this training internship, we used the features the network learned, which we call “fingerprints,” to predict ER, PR, and Her2 status in two datasets. Despite the discovery dataset being relatively small by the standards of the machine learning community (n = 939), fingerprints enabled the determination of ER, PR, and Her2 status from whole slide H&E images with 0.89 AUC (ER), 0.81 AUC (PR), and 0.79 AUC (Her2) on a large, independent test set (n = 2531). Tissue fingerprints are concise but meaningful histopathologic image representations that capture biological information and may enable machine learning algorithms that go beyond the traditional ER/PR/Her2 clinical groupings by directly predicting theragnosis.

## Introduction

Although deep learning (DL) has potential to teach us novel aspects of biology, the most impressive use cases to date recapitulate patterns that experts already recognize^1–6^. While these approaches may improve inter-observer variability and accelerate clinical workflows, our goal is to use DL to learn how morphology from hematoxylin and eosin (H&E) images can be used to predict biomarkers^7^, prognosis^8^ and theragnosis—tasks which are not currently possible for a pathologist to do by eye, but if possible, could improve our understanding of cancer biology. However, since DL learns from data, it needs large training sets to learn patterns. One of the biggest challenges is obtaining large, well annotated training sets.

While typical computer vision datasets contain on the order of millions of annotated images to learn statistically significant relationships^1^, clinical pathology case sets generally number in the hundreds. Moreover, noise in the clinical annotations dilutes the learning signal and increases the probability the network will learn spurious features like stain color, clinical site, or other technical variations^9–11^. To overcome this limitation, we developed the concept of “tissue fingerprints,” based on the hypothesis that molecular differences of the tumor are often translated into subtle differences in morphologic phenotypes. This idea is akin to the paradigm of precision medicine, where instead of grouping patients, individual patients are treated based on their specific molecular and environmental parameters. Hence, instead of training a network to distinguish between groups of samples, we first pre-configure the network to recognize, or “fingerprint,” individual tumors, a task which can leverage large, unannotated datasets, which are widely available. After pretraining a network to fingerprint tissues, we expect that a smaller amount of annotated data will be necessary to adapt it to a clinical task.

**Figure 1:**
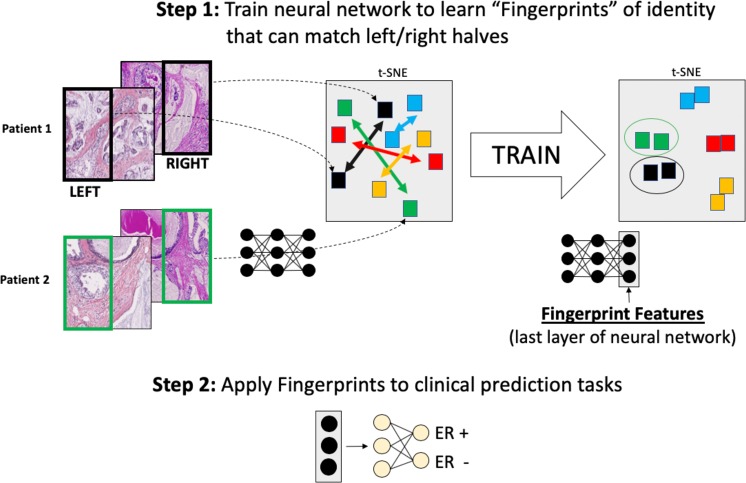
Networks are first trained to learn tissue fingerprints, which are patterns of cells and tissue visible on H&E images that can be used to distinguish between patients. Following this training internship, which can be scaled to very large numbers of patients without clinical outcome annotations, the fingerprints are repurposed to make clinically relevant predictions from small labeled datasets.

To implement tissue fingerprints, we trained a neutral network to fingerprint pathologic tumor samples from a training set and then tested it on a simple matching task using tumor images from new patients. Briefly, multiple images were divided into halves, and a network attempted to learn a vector of features (a “fingerprint”) that could correctly pair the halves (Fig. 1). An important aspect of this work was using the matching task to learn stain- and site- invariant features of architecture. We controlled for these sources of noise by testing whether the fingerprints could match tissues from the same patients that had been stained and scanned at different sites. Optimizing on the task of matching, we performed experiments testing the impacting of training set size and methods of image normalization.

Once this training internship was accomplished, the features (fingerprints) were extracted from the pre-wired fingerprint network and used to classify between groups of tumors with biologically relevant molecular pathway annotations^12,13^. We looked at clinical estrogen receptor (ER), progesterone receptor (PR), and Her2 status in breast cancer, which are important predictive and prognostic molecular markers currently assessed by molecular immunohistochemistry (IHC) staining^14^. We tested whether a fingerprint-based hematoxylin and eosin stain (H&E) classifier could predict this molecular information from tissue architecture and visualized the regions that classifier used to reach a prediction.

## Methods

### Tissue fingerprinting

#### Dataset

The tissue fingerprint network was trained on images of tissue microarray (TMA) cores. The tissue microarrays (TMAs) used in this study were obtained from supplier US Biomax, Inc. Array BR20823, containing 207 tissue cores from 104 patients, was used to train the fingerprint network. Array BR20819, containing 208 cores from a separate group of 104 patients, was used to test the trained model. We obtained one or two sections from each array (for BR20823 and BR20819, respectively), which were H&E stained using standard laboratory protocols, before scanning was performed at 40x resolution (0.249 microns per pixel) on a Carl Zeiss slide scanner. US Biomax, Inc. kindly provided us with images of serial sections of these microarrays that had been stained and scanned by their protocols on an Aperio slide scanner at lower resolution (0.49 microns per pixel). Additional breast cancer tissue images were used to increase the size of the training set for experiments 2 and 4. These images are of breast cancer tissue from a variety of sources having distinct patients from BR20819 and BR20823.

#### Neural network training (experiments 1 and 2)

Neural networks were trained and run on NVIDIA P100 GPUs (Oracle Cloud Infrastructure BM.GPU2.2 instances). The fingerprint network was trained on image patches randomly extracted from the BR20823 images. Each circular tissue core was isolated, scaled to 0.5 micron/pixel resolution (bilinear resampling), and cropped to a 1600 × 1600 pixel square. These squares were assigned a numeric index from 1 to 207, reflecting their position on the array. Each square was then divided into left and right halves. During training, a small image patch (224 × 224 px) was sampled from the left half, augmented through rotation, color spectrum augmentation^1^, color normalization^6^. It was then passed to a neural network trained to minimize cross entropy loss between patient identity and a predicted index. During training, we monitored progress by measuring how well the network could predict the core index from patches from the right halves of the tissue images, which it hadn’t seen. When this accuracy plateaued, we stopped training and tested the quality of the features on the tissue matching game. Experiments 3 and 4 used the more complex loss function described below.

In the four experiments described, we used the standard implementation of the Resnet34 architecture^15^ provided by the PyTorch library^16^. The network was randomly initialized and trained from scratch. Additionally, we trained larger networks, including Resnet50 and Resnet100, using the conditions of experiment 4, but found the same performance as Resnet34 (results not shown). The benefit of larger networks may depend on the size of the training dataset. Here we demonstrate the concept of fingerprinting with a relatively small dataset, but training on larger datasets may demonstrate added benefits of deeper networks.

#### Promoting style invariance by GAN-based style transfer (experiments 3 and 4)

**Figure 2:**
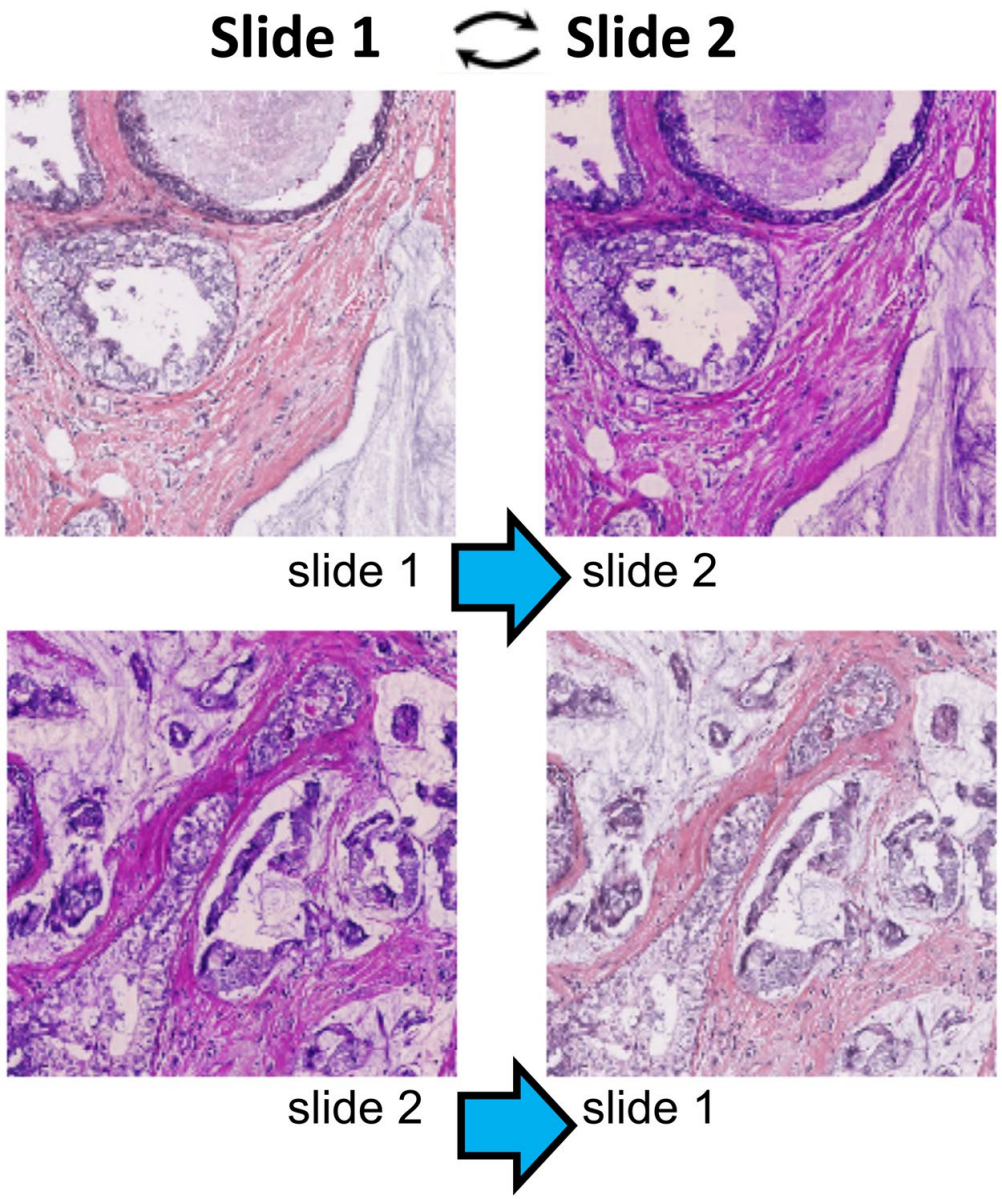
CycleGAN normalizes the staining colors and styles of tissue images while preserving morphology. Top: the style of slide 1 is translated into the style of slide 2. Bottom: the style of slide 2 is translated into the style of slide 1.

Neural style transfer was performed offline using CycleGAN^17^, a method of neural style transfer, that aims to alter the style of images while preserving fine details. Briefly, the CycleGAN approach consists of training two neural nets, a generator which takes an image A transforms it into an image of style B, and a discriminator which is trained to distinguish between generated images and real ones. The networks are trained simultaneously as adversaries. As the discriminator improves, the generator is challenged to learn better transformations from style A to B. Conversely, as the generator improves, the discriminator is challenged to learn better features that distinguish real and generated images. In this project, we used the open-source CycleGAN code without modification. We trained the network to transfer styles between images of BR20823 that were stained by the array manufacturer (US Biomax) or at our site (style transfer between slides 1 and 2, respectively, as shown in Fig. 2). Thus, our original set of 13,415 cores was augmented to three-fold its original size via neural style transfer (each core has an original image, a virtual USC stain and a virtual Biomax stain). Following style transfer, we adapted the loss function to promote style invariance. The new loss function has two components, a cross entropy loss (abbreviated ‘CE’) to predict the identity of each patch, which was the loss term used in experiments 1 and 2, plus an additional loss term to minimize the distance of fingerprints from different styles. The additional loss term is the squared error (abbreviated ‘SE’) between the L2-normalized fingerprints, where a fingerprint is the 512-dimensional (512D) feature vector from the last layer of the Resnet.

Loss is defined for a pair of images from tissue core_i_ (1 ≤ i ≤ 207): Image_i1_, and Image_i2_. Image_i2_ is a re-styled version of Image_i1_ that contains the same morphological information. The loss is a sum of two cross entropy losses and a fingerprint distance loss. The symbol, $\gamma$, is a constant. In our experiments, we used $\gamma$ = 0.5.

$$loss(Imag{e}_{i1},Imag{e}_{i2},y=i)=CE(Imag{e}_{i1},\,y)+CE(Imag{e}_{i2},\,y)+\gamma (FPdist(Imag{e}_{i1},Imag{e}_{i2}))$$

Cross entropy is defined using the classification vector produced from the network for each image. This vector contains 207 elements. Image_ix_ from core_i_ produces a classification vector c_ix_.

$$CE(Imag{e}_{ix},\,y=i)=-\,\mathrm{ln}\left(\frac{{e}^{{c}_{ix}[y]}}{{\varSigma }_{z}\,{e}^{{c}_{ix}[z]}}\right)$$

Fingerprint distance loss is defined using the fingerprints produced by the neural network for each of the images. If the fingerprints for Image_i1_, and Image_i2_ are called FP_i1_ and FP_i2_, fingerprint distance loss is the following. || || and SE refer to the L2 norm and the Euclidean distance, respectively.

$${d}_{FP}^{2}(Imag{e}_{i1},Imag{e}_{i2})=SE\left(\frac{F{P}_{i1}}{||F{P}_{i1}||+{\epsilon }},\,\frac{F{P}_{i2}}{||F{P}_{i2}||+\,{\epsilon }}\right)$$

#### Creating heat maps of recognized regions

We generated heat maps of tissue cores showing the parts of an image most predictive of tumor identity (Fig. 4). The region colors were determined as follows: each core image was divided into overlapping square patches (size 224 × 224 pixels, with 80% linear overlap). Each patch was passed to the neural network, and a probability vector was calculated predicting the identity of each core via Softmax. Since there are multiple cores per patient, we aggregated the 207 probabilities into 104 probabilities (one per patient) by summing the probabilities of cores that came from the same patient. Each heat map shows the probability of predicting the correct patient and is shaded from 0 (blue) to 1 (red).

### ER, PR, Her2 Classification from whole slides

#### Datasets

The whole slide images used in this study were obtained from The Cancer Genome Atlas^12^ (TCGA) and the Australian Breast Cancer Tissue Bank^13^ (ABCTB). We included 939 cases Breast Carcinoma from TCGA and 2531 Breast Cancer cases from the ABCTB. Clinical characteristics are summarized in supplemental Table 1. Of note, molecular status of ER, PR, and Her2 was assessed clinically, where annotations were made on the patient level.

**Table 1:** Fingerprinting Datasets.

Tissue Microarray Slides	Slide 1	Slide 2	Slide 3	Slide 4	Slide 5
Section name	BR20823_BM	BR20823_16	BR20823_17	BR20819_BM	BR20819_42
TMA section number	27	16	17	84	42
*n* patients from 20823	104	104	104	0	0
*n* patients from 20819	0	0	0	104	104
*n* cores	207	207	207	208	208
Site performing H&E stain	US Biomax Inc.	USC	USC	US Biomax Inc.	USC
Original scan resolution	0.5 µm/pixel	0.24 µm/pixel	0.24 µm/pixel	0.5 µm/pixel	0.24 µm/pixel
					

#### Training a patch-based ER,PR,Her2 classifiers directly from images (control)

Similar to other deep learning approaches, we trained a patch-based classifier to predict molecular marker status. All experiments were conducted using five-fold cross validation. First, each whole slide image was grossly segmented into foreground vs. background. The foreground areas were divided into non-overlapping squares of 112 × 112 microns, which were scaled to a final patch size of 224 × 224 pixels. 120 patches per patient were randomly selected and used for downstream analysis. To train the classifier, the entire set of TCGA patients was split into five groups. For each cross validation fold, three groups were used to train, one group (the “overfitting group”) was used to monitor overfitting and perform early stopping, and the remaining group was used to test the network’s final performance. To the train the network, patches were assigned a binary label per the patient-level annotation, but early stopping was implemented by averaging the predictions of all patches belonging to overfitting group and measuring the patient-level AUC (area under the ROC curve) score. These experiments used a standard implementation of Resnet34.

#### Training ER, PR, Her2 classifiers directly from fingerprints

Using the same image patches and cross validation splits from the control experiment (described above) and the previously trained fingerprint network, we extracted 512D fingerprints for each image patch and then trained a second “biomarker” neural network to predict marker status based on these fingerprints. The second network has the following structure: input, 512 × 8 linear layer, rectified linear unit nonlinearity, 8 × 1 linear layer, hyperbolic tangent nonlinearity. The parameters of this network were trained in batches consistent with multiple instance learning (see Supplemental Methods). Similar to the control experiment, multiple patch predictions were pooled to make patient-level predictions, and the reported ROC curves compare patient-level score and clinical marker status. As a control for the features, we also implemented the same workflow using image features extracted from a Resnet34 network pretrained on the ImageNet dataset. The parameters for this model were obtained from the torchvision Python library.

#### External validation of the whole-slide ER, PR, or Her2 classifier

After the five-fold cross validation experiments on the TCGA dataset, we validated our classifier on an independent test set from the ABCTB. These images were processed like those from TCGA: 120 patches (112 × 112 microns, resized to 224 × 224 pixels) were extracted per patient, and fingerprints were calculated using the pre-trained fingerprint network. We then used the TCGA-trained biomarker network to predict marker status of patients in the ABCTB dataset. We calculated an AUC score from the ROC curve comparing the neural network predictions to clinical status from the ABCTB.

### Visualization of regions used to predict clinical ER score

#### Generating heatmaps of predicted ER status

Given the high AUC for the ER classifier, we sought to make heatmaps showing the regions predicted to be highly ER-positive or negative across whole slides. We extracted every non-overlapping foreground patch (112 ×112 micron) from TCGA WSIs and compressed them into a 512D fingerprints using the pretrained fingerprint network. Subsequently, we used the pre-trained ER network to make patch-level predictions across the entire slide. The predictions are shaded in grayscale. Black signifies a prediction of −1 (ER-negative), while white signifies +1 (ER-positive). Gray values correspond to scores close to 0 (indeterminate). All visualizations of ER predictions and features show the validation and test sets of one cross-validation split. Hence, none of the data reflect images that were used to update the values of the weights.

#### Heatmaps of tissue types

TCGA whole slides were segmented into background, epithelium, stroma, and fat by training a patch-based classifier on a publicly available dataset of WSIs with tissue type annotations^18^. The segmentations were subsequently assessed for gross accuracy at 2x resolution, and a small portion of heatmaps (<5%) were manually corrected.

#### Visualizing the tSNE embedding of WSI fingerprints

We used an efficient implementation of the tSNE algorithm to plot a 2D manifold of the fingerprints^19^. Each point represents one fingerprint, which corresponds to a single patch from a WSI. The color of the point represents the ER prediction, which is shaded from blue (−1, ER negative) to green (0, neutral) to red (+1, ER positive). Grossly, we appreciated the presence of regions containing a predominance of blue or red points. To assess the visual similarity of patches in these clusters, we manually selected 12 cluster centers and visualized five patches closest to these centers.

## Results

In summary, the basic aim of this project was to develop a biologically meaningful set of H&E histologic features. We hypothesized that in the process of training a neural network to “match two halves” of tissue cores, it would learn a compact, but meaningful representation of tissue architecture. Following training of the fingerprint network, which was influenced by the discovery of the importance of stain-normalization, we compared various methods of predicting molecular marker status from whole slides. We found that a fingerprint-based approach out-performed traditional transfer-learning and direct patch-based classification. Moreover, fingerprint-based classifiers continued to perform well on an independent, external dataset. When we applied the fingerprint-based classifier to a small collection of slides in the test-set, we found that they produced interpretable heatmaps, and predominantly focus on epithelial patterns to make predictions.

### Learning fingerprints

We trained the networks to learn fingerprints from tissue cores in TMAs. The TMA format makes it easy to process one set of tissues in multiple ways and allowed us to simulate the batch-effects that are commonly encountered at pathology labs. By staining and scanning one section at USC and another stained by the TMA supplier (US Biomax), we obtained paired images with the same architectural features, but different coloration. Our goal was to learn a fingerprint that summarized the architecture but ignored the staining differences.

We used one TMA to train (BR20823) and another TMA to test (BR20819, Table 1). Each TMA contains approximately 208 tissue cores from 104 patients, with no patient overlap between arrays. We used 3 serial sections of the training TMA. One section was stained/scanned by the TMA supplier (slide 1), the other two were stained at USC (slides 2, 3). We similarly collected 2 serial sections of the test array, stained at USC and by the TMA supplier (slides 4 and 5).

### Stain normalization is necessary for fingerprinting

We performed four experiments, varying training set size and image normalization, to determine how to best train a fingerprint network (Table 2). We hypothesized that training on large numbers of cores would improve accuracy, and that image color normalization would greatly improve training on small data but have a smaller effect on larger datasets. In experiment 1, the baseline, we collected the 207 tissue cores from slides 1 and 2 (serial sections of the training TMA that were stained by the supplier and USC, respectively), divided them in half, and trained the network to recognize patients based on patterns in one of the halves. (We arbitrarily chose to train on the left halves). Each core was assigned a number from 1 to 207, and the network was trained to identify the number from a patch sampled from the image-half. In experiment 2, we scaled the dataset over 20-fold: adding 13,000 additional training images. Again, we trained the network to predict the index. In experiments 3 and 4, we used the same datasets as before, but included a color normalization procedure based on neural style transfer^17,20^. In the first two experiments, we predicted that, as the network was trained to recognize increasing numbers of images, it would automatically learn stain-invariant features. In the second two experiments, we used the style transfer algorithm CycleGAN^17^ to recolor images (Fig. 2), making them appear as if they were prepared at a different site. CycleGAN can exchange the texture between two spatially similar image sets, while preserving overall structural information. Compelling examples include transforming photographs into impressionist paintings and horses into zebras. Here, we use CycleGAN to transfer the H&E staining coloration from a reference site to images from other sites. Then we trained the networks to look at both images and predict the same features, cancelling out the effect of stain variation.

**Table 2:** Fingerprinting Results.

#	Description	Dataset Size N. Training Cores (N. Patients)	Neural Style Transfer	Network	# Test Cores	CV Acc.	Test: Core Acc.	Test: Patient Acc.
1	Small Dataset/No Style Transfer	414 (104)	−	Resnet 34	208	77%	22%	−
2	Large Dataset/No Style Transfer	13415 (3302)	−	Resnet 34	208	77%	26%	−
3	Small Dataset/Style Transfer	414 (104)	+	Resnet 34	208	93%	43%	−
4	Large Dataset/Style Transfer	13415 (3302)	+	Resnet 34	208	95%	63%	93%

To compare the quality of the fingerprints learned in the four experiments, we performed tissue matching on the test TMA sections. Using the thus-trained NN, we calculated fingerprints for left halves of cores from one section (stained by the array manufacturer, slide 4) and the right halves from the other (stained at USC, slide 5), and matched each left fingerprint to the nearest right fingerprint in 512D fingerprint space. Since there were 208 cores in the test set, we report a core-level accuracy (acc. = number of cores matched correctly/208). The null accuracy by chance is 0.4% (1/208 cores).

While fingerprints from all four experiments matched cores better than chance, the accuracy was highest in experiment 4, which used a large training set with stain normalization. The fingerprints from this method matched cores with 63% accuracy (131/208 cores). Surprisingly, stain normalization seems to be necessary to get the performance gains of larger training sets. Comparing the results of experiments 2 and 3 to the baseline, increasing training set size in the absence of stain-normalization (experiment 2) provided only a miniscule improvement in matching accuracy over the baseline. However, stain-normalization nearly doubled the accuracy. It’s important to note that in all four experiments we used standard image augmentation during training. Immediately before the image was shown to the fingerprint network, it was randomly adjusted for brightness and contrast and converted to grayscale. Even with these procedures, which were intended to make networks invariant to color differences between images^6^, doing an additional style normalization step before the augmentation provided a significant improvement.

When we examined the mistakes from the network in experiment 4, we noticed that a large portion of them were due to an aspect of the study design, using a test set with 2 cores per patient. Several of the misclassified cores were incorrect at the core level but correct at the patient level. This is because some tumors are morphologically homogeneous and have similar patterns across cores. Thus, we also calculated a pooled accuracy, which uses the fingerprints from both left cores to match both right cores and found that fingerprints could match patients with 93% accuracy (see methods for details).

Encouraged by the high patient-level accuracy of the matching, we started studying the features of the final neural network layer. When the network is shown an image, this layer produces a 512D vector of real numbers, the tissue fingerprint. In the remainder of the work, we apply the network to extract fingerprints and explore how they can be used to link histologic patterns to clinical subgroups of breast cancer.

### Fingerprint visualizations reveal style-invariant histologic patterns

**Figure 3:**
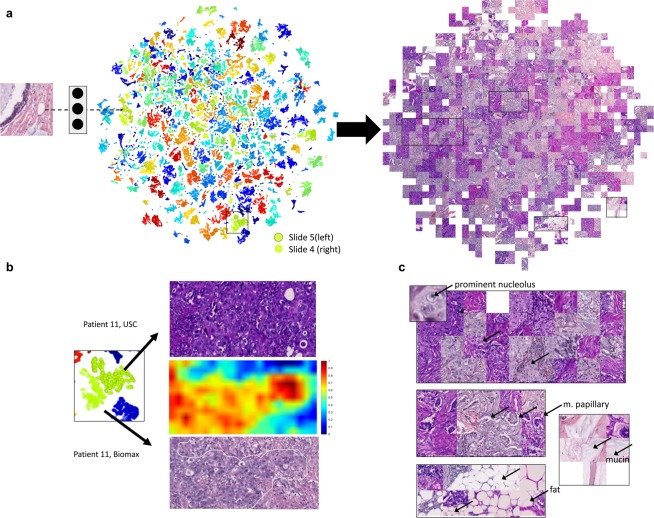
(**a**) Representative tSNE visualization of fingerprints from the test set. In this visualization, left halves from slide 5 and right halves of slide 4. (**b**) Visualization of a representative pair. Left half presented on top, right half on the bottom, middle shows a heat map of fingerprint distance (distance from fingerprints from the bottom image to the average fingerprint of the top image). (**c**) Left, exploded displays of the original patches in the embedding show similar histologic features (nucleoli, micro-papillae, fat, mucin).

As both a control and as a starting point for understanding the features in the fingerprints, we performed two-dimensional fingerprint visualization. We took the left and right halves of cores from the test slides, which had been stained at different sites, and calculated fingerprints for patches from the halves. Next, we embedded the fingerprints in a tSNE plot (Fig. 3a). tSNE is a technique that compresses high dimensional vectors into a 2D space while preserving local structures in the data^21^. Its primary utility in this work is to approximate the relationships within a high dimensional space and make them accessible for visual analysis. After using tSNE to compress the fingerprints into 2D coordinates and plotting each coordinate as a point colored by patient index, we observed that the left and right halves from the same patient are close in the embedding space. Since points that are nearby on a tSNE plot also tend to be nearby in the original space, this visualization provides evidence that fingerprints of similar tissues are similar in spite of differences in staining. Moreover, visualizing the same embedding as a map of image patches, instead of colored points, reveals that different regions of the map contain different architectures, such as nucleoli, fat, micropapillary growth, and mucin patterns (Fig. 3c). Thus, in the embedding space, fingerprints are clustered by histologic patterns even if the patches they come from exhibit markedly different colorations and styles.

### Fingerprints can be used to visualize similar regions between tissues

In Fig. 3b, we focus on a specific Left/Right pair from the test set. We calculated the average fingerprint of the right half and plotted a heat map showing the similarity (defined as 1 - normalized Euclidean distance) from each patch in the left half to the average fingerprint of the right half (red is similar, blue is dissimilar). The overlay (bottom) shows that similarity between the right and left halves is highest in a discrete region that appears to contain epithelial cells. This observation is consistent with the abundance of epithelial cells in the right image, suggesting that fingerprint similarity may have utility in histologic search.

### Fingerprints combine epithelium and stromal features

**Figure 4:**
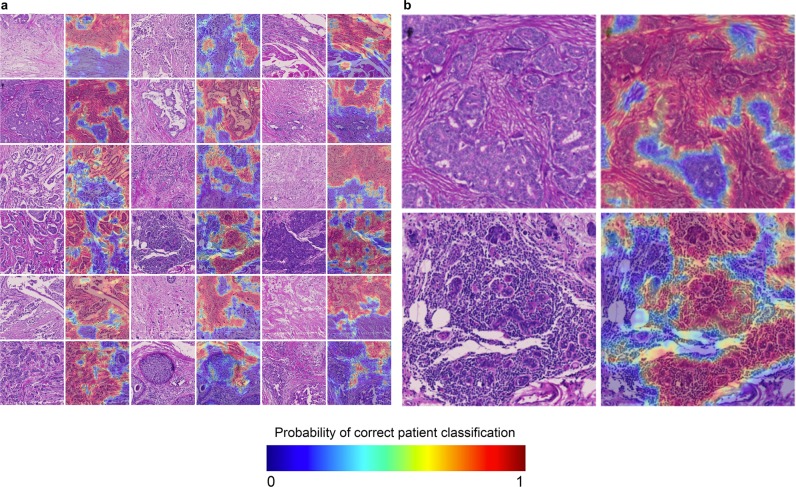
(**a**) Heat maps of areas that lead to accurate patient classification. Higher probability regions (red) are more predictive of patient identity, and hence distinctive, than blue regions. (**b**) An exploded view of two cores from (**a**).

To directly visualize the image components comprising its fingerprint, we generated heat maps of tissue cores, highlighting image regions that most accurately predict patient identity (Fig. 4a). We show the original H&E images alongside heat maps of patient prediction accuracy using corresponding image regions. Red areas identify the patient accurately, and blue ones do so poorly. Based on the presence of both red and blue areas, some core regions are more predictive of patient identity than others, meaning their patterns are specific to that patient’s tumor. Figure 4b shows an exploded view of two cores. The red-colored regions demonstrate the classifier looks at a combination of stromal and epithelial areas.

### Fingerprints relate to molecular status of breast cancer

Because each tumor has unique genetic and microenvironmental interactions, we hypothesized that networks trained to recognize patients would implicitly learn features that reflect the underlying biological processes. For breast cancer, ER/PR/Her2 status is among the most important indicators of prognosis. Hormone-receptor (ER/PR) positive tumors tend to be less aggressive and occur in older patients. Additionally, ER-positive tumors can be treated effectively with drugs that target the estrogen axis. Similarly, Her2-positive tumors can be treated with drugs that target the Her2 axis. For these reasons, the NCCN task force mandate^22^ that ER, PR and Her2 status be measured for every new case of breast cancer.

**Figure 5:**
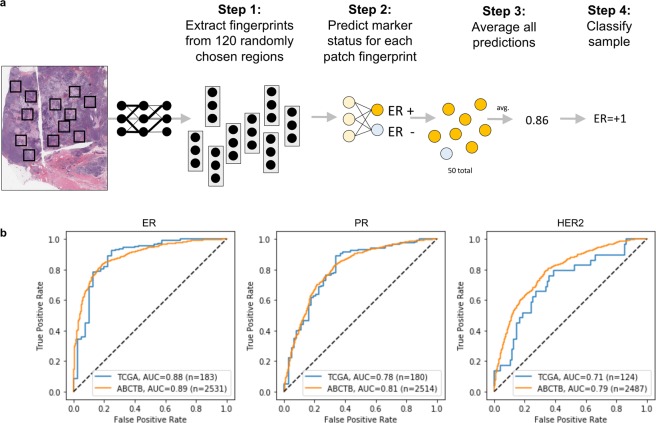
(**a**) Illustration of whole slide clinical ER classification. An analogous procedure was used for PR and Her2 classification. Fingerprints were extracted from 120 random image patches, and a second ER-classifier, acting on the fingerprints, made local predictions, which were averaged to produce a continuous whole-slide-level ER-score. (**b**) Receiver operating characteristic curves (ROC) for clinical ER (left), PR (center), and Her2 prediction (right). The TCGA ROC curve reflects a test set from five-fold cross validation, and the AUC corresponds to the average area under the ROC curves of all five TCGA test sets. All samples in the ABCTB dataset are test samples and were never seen during training. Sample sizes vary depending the availability of clinical annotations.

While ER, PR, and Her2 status is routinely assessed by immunohistochemistry (IHC) staining for the receptors (Her2 can also be measured by FISH staining), we explored whether H&E morphology, quantified via fingerprints, could serve as a surrogate marker of these proteins. Initially, we queried this hypothesis on a set of breast cancer images curated TCGA^12^. These samples consist of H&E whole slide images (WSIs) from 939 patients at 40 sites. First, scaled the images to 20X resolution, randomly extracted 120 image patches per image, and fed them through the fingerprint network to calculate fingerprints (Fig. 5a, step 1). Then, we trained a second neural network to compress the fingerprints (512D vectors) into a patch-wise prediction score of a particular receptor from −1 to +1 (Fig. 5a, step 2). For simplicity, the figure indicates the process for predicting ER; however, the same procedure was used for PR and Her2. Finally, we averaged the predictions across the image to estimate the patient’s receptor status (Fig. 5a, steps 3-4). We trained on the TCGA dataset with five-fold cross validation. The patients were split into five groups: three were used to train the second network; one was used to monitor training progress and decide when to stop training; the remaining group was tested. The plot in Fig. 5b (left) shows the ROC curve for a representative test set from the TCGA data, for ER classification. The average AUC of the test sets was 0.88 (n = 183, per test set). This is the highest ER classification score we have observed, including our previous work using nuclear morphometric features (0.72 AUC)^23^ and other recent works that predict molecular characteristics of breast cancer from H&E tissue microarray images^24–26^. To validate these findings, we obtained WSIs of 2531 breast cancers from the ABCTB^13^, and tested whether the TCGA-trained classifier could predict ER-status in this group. We measured an AUC of 0.89 on this dataset (n = 2531). Applying the same pipeline to PR and Her2, we found that fingerprints could predict PR in the TCGA dataset with an average test set AUC = 0.78_(n = 180, per test set), and AUC = 0.81 (ABCTB, n = 2514) (Fig. 5B, center and left). The results for Her2 were AUC = 0.71 (TCGA, n = 124) and AUC = 0.79 (ABCTB, n = 2487). As a methodological control, in addition to these experiments, we trained a classifier to predict ER status directly from image patches and measured an AUC = 0.82 (TCGA, n = 138).

### The fingerprint-based ER classifier learned epithelial patterns

**Figure 6:**
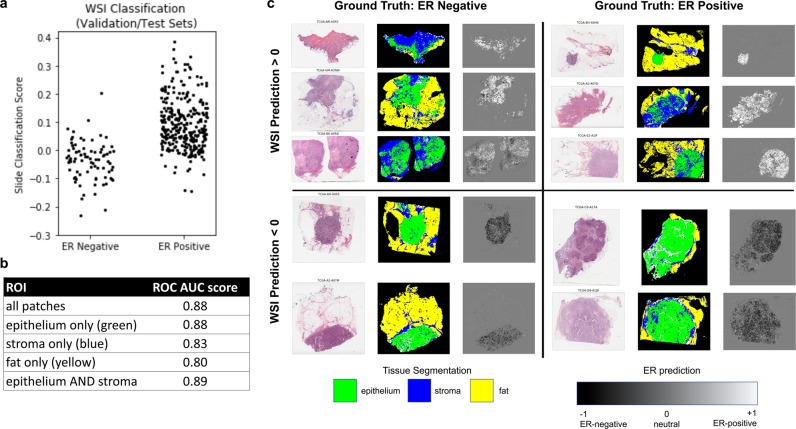
(**a**) Histogram of ER-predictions from the TCGA test set averaged across the entire slide (AUC = 0.88). (**b**) AUC scores obtained by pooling ER predictions from different regions within slides. (**c**) Representative heatmaps of correctly and incorrectly classified whole slides. WSI prediction was obtained by averaging over all patches (epithelium, stroma, fat). Each slide visualization consists of an RGB thumbnail, a tissue type segmentation, and an ER prediction heatmap.

To understand the regions that were used to make biomarker predictions, we applied the trained ER to whole slides. Our first question was whether the ER classifier worked best on epithelium vs. stroma vs. fat. Briefly, we divided each whole slide image into non-overlapping patches and performed image segmentation to classify each patch as epithelium, stroma, fat, or background. Additionally, we calculated a 512D tissue fingerprint for each patch and made ER predictions for each patch. When the patch-predictions were averaged across the entire slide, they were able to classify ER with an AUC of 0.88 (histogram shown in Fig. 6a). We also subset the patches by tissue type and calculated the AUCs after averaging patches of different types (Fig. 6b). We found that the classifier was most accurate when we pooled predictions from epithelium patches, followed by stroma, followed by fat. Moreover, pooling across epithelium only, or epithelium and stroma was essentially equivalent to pooling across all patches.

These findings were visually supported by heatmaps generated from the slides (Fig. 6c). We present representative slides that were correctly and incorrectly classified: H&E thumbnails, tissue type segmentations, and ER-prediction heatmaps shaded from black to white representing a prediction of −1 to +1. We observe that while most areas in the slide have a score of approximately 0 (gray), regions that are strong predictors (white or black) tend to lie in epithelial regions (green). Even when the predictions are inaccurate, the classifier makes mistakes based on misclassifying epithelial regions. For instance, false positive and false negative slides, shown on the upper-left and lower-right quadrants of Fig. 6c show strong white and black signal in epithelial regions.

### A low dimensional embedding of fingerprints reveals histologic patterns that predict ER status

**Figure 7:**
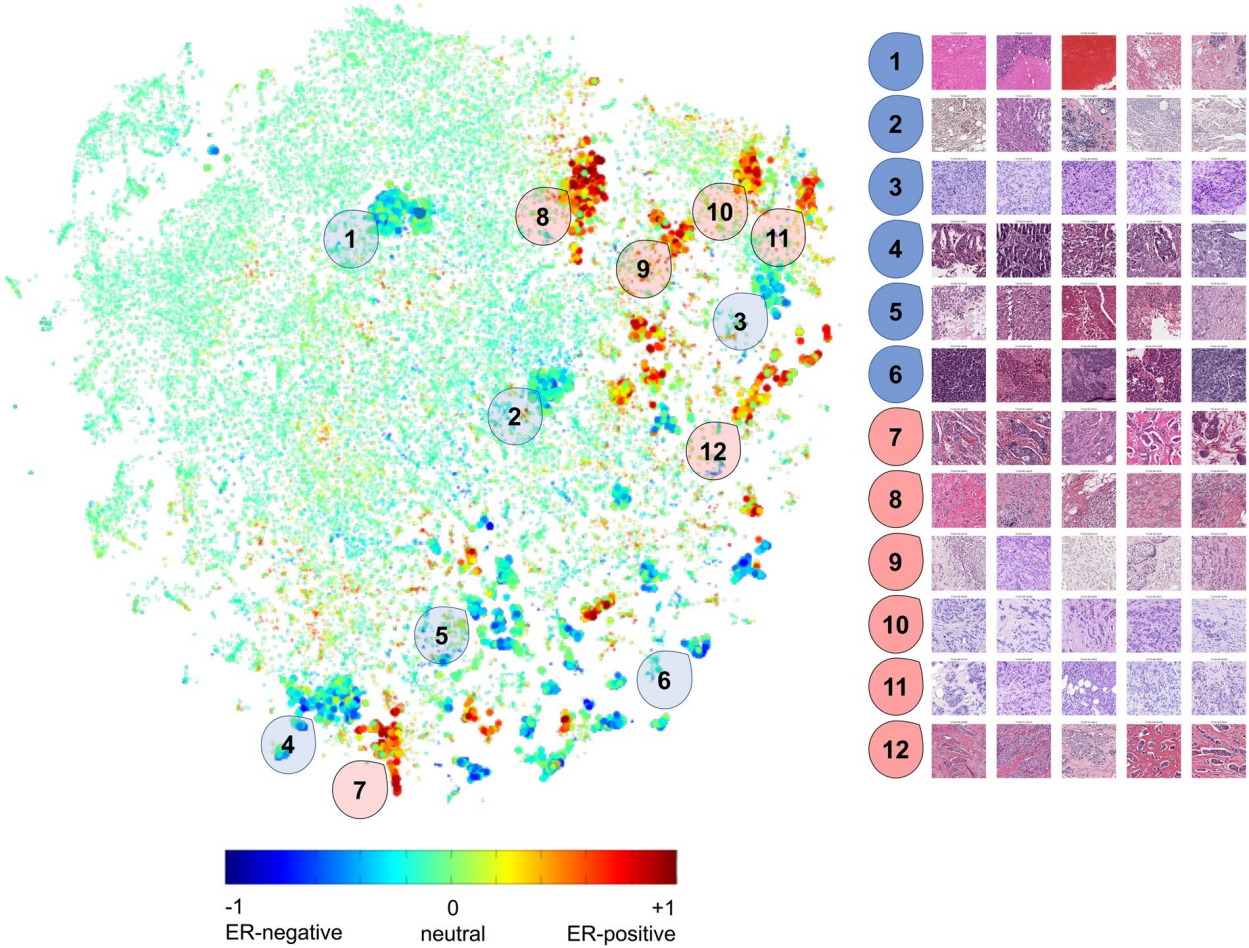
Left: tSNE embedding of fingerprints from patches extracted from TCGA whole slides, shaded by ER prediction score. 12 clusters with high positive or negative enrichment were selected for manual inspection. Right: H&E patches closest to the cluster centers. Each patch is from a different patient. High resolution image in supplemental information.

To test the hypothesis that tissues predicted to be ER-positive or negative may have similar visual characteristics, we embedded fingerprints from whole-slides into a low dimensional space using a fast implementation of the tSNE algorithm. tSNE is an unsupervised non-linear technique which compresses high dimensional vectors into a low dimensional space, while preserving local structures in the data. In our case, we used tSNE to compress a matrix of fingerprints (512D) into a 2D embedding space (Fig. 7). Each point in this embedding represents a fingerprint from a single patch, and there are 120 patches (points) per WSI on this plot.

When we shaded the points by the ER score predicted by the ER-classifier, we noticed that most points fail to strongly predict ER status (colored green, prediction approx. 0). However, there were several noticeable clusters of points predicted to be ER-negative (blue) or ER-positive (red). Of these clusters, we manually selected 12 areas to explore (six ER-negative, and six ER-positive), and present the five patches from each cluster. Of note, each patch is from a different patient (detailed image in supplemental data). Examination of the image patches reveals shared histologic features within each cluster. For instance, cluster 1 reveals regions of necrosis and relatively sparse cells. Patches in cluster 2 include patches with large quantities of immune cells, and cluster 7 contains nests of tumor cells.

## Discussion

Deep learning is transforming computer vision and cancer pathology. As training sets scale, there are substantial increases in accuracy on diagnosis, prognosis and theragnosis^27^. However, the biggest gains are likely to come when we learn to leverage a greater spectrum of available pathology images, including the vast majority of images which are mostly or completely unlabeled. Here, we illustrate a novel first step, using tissue matching to discern features that are distinctive for a patient but differ between individuals. While tissue matching is not a skill formally taught in pathology training, it allows a neural network to discover key discriminating histologic features from a large set of unannotated images. Interestingly, these discriminatory features, or fingerprints, tend to reside at the interfaces between epithelial and stromal cells, and may reflect tissue specific genetic and microenvironmental parameters. While this study used serial sections of a TMA to design a rigorous implementation of core-matching, the resulting network trained in experiment 4 demonstrates that a training paradigm that incorporates style normalization may benefit significantly from histology images of any type, not just matched TMA images.

Training a network on the task of tissue identification also improves the interpretability of DNNs and provides insights about the elusive “black box” of deep learning. The ground truth (tissue identify) is indisputable, and visualizations reveal cohesive, biologically interpretable patterns that leverage parameters which are likely to reflect unique underlying genetic and microenvironmental interactions. Hence, we anticipate that fingerprints, which identify features that discriminate between individuals, will be useful when applied to tasks that seek to discriminate between groups of biologically different tissues.

Our experiments demonstrate the significant predictive power of such fingerprints to predict the molecular status of a tumor. Taking the fingerprint network, we extracted fingerprint features from whole slide images and used them to predict ER, PR, and Her2 status from two independent breast cancer cohorts. We initially trained and validated our algorithms on images from The Cancer Genome Atlas (TCGA), with cross-validation. Then, performed independent validation on samples from the Australian Breast Cancer Tissue bank (ABCTB, n = 2351) achieving the following areas under the curve: 0.89 (ER), 0.81 (PR), and 0.79 (Her2). These metrics are higher than all previously published attempts to predict molecular information from H&E images. The improved performance is secondary to the implementation of tissue fingerprinting. The performance we found is similar to previous studies assessing the correlation between IHC and microarray assessments of ER and PR, which found good concordance between frozen and IHC for ER (93%) and lower for PR (83%)^22,28^. We believe that using tissue fingerprints will ultimately enable direct treatment response prediction in breast and other cancers, to an accuracy above that provided by current molecular approaches.

While classification accuracy is an important metric for an algorithm’s utility, the significance of fingerprinting extends beyond this because it enables the interpretation of the histologic patterns learned by deep neural networks. At present, if interpretability is the goal, deep learning is not necessarily the best approach. Using human designed, handcrafted, pre-extracted features such as cell shape, cell neighborhood statistics can provide rapidly interpretable insights about the tissue properties that correlate to a clinical or molecular outcome^29,30^. However, the downside of these approaches is introduction of human bias and the challenge of building workflows to accurately extract these features.

While the flexibility and automaticity of deep learning makes it effective for black box usage in a number of scenarios, interpretability is essential for developing testable hypotheses that advance basic biomedical research. Thus, we were encouraged by the interpretability of our fingerprint-based ER classifier. The prediction heatmaps shown in Fig. 6c demonstrate that the network learned to make positive, negative, and neutral predictions. Thus, it automatically learned to ignore some regions (e.g. neutral, gray areas), while paying attention to others. In this case, it learned to pay attention to areas of tissue epithelium.

A second insight came from plotting the tSNE embedding of fingerprints and discovering natural clusters of patches with similar histologic characteristics predicted to be ER-positive, ER-negative, or neutral (Fig. 7). There is a significant history of histological classification of breast cancer patterns. Numerous attempts have been made to develop histologic typing schemes, but these are subjective, difficult to reproduce, and a large number of slides frequently fall into the “no specified type” category (lacking distinctive characteristics of any pre-defined type). The embedding we present in Fig. 7 provides preliminary support for the development of machine-automated histologic typing, in which tissue patches are clustered by visual/histologic similarity. Of note, our algorithm learned this embedding without knowing the molecular status of the tissues, and the embedding is not sensitive to differences in staining colors. Patches in these clusters have different shades of pink and purple H&E but share core histologic features.

The observation that some of the clusters seem to correlate with ER status suggests a link between tissue architecture and clinical ER status. This view suggests that only a subset of patches/tissue architectures contain useful diagnostic information for making this prediction. Future work will determine whether predicted fingerprints can be used to make biomarker predictions while providing confidence scores based on the quantities and types of tissues present in the slide. Assigning a confidence score to a prediction may facilitate triage and workup of the most relevant biological markers for a particular patient.

An additional area to focus on is how these visualizations can be used to improve a classifier with intermediate accuracy. A potential limitation of this approach is that we demonstrate its application with respect to ER classification, but not PR or HER2. We made this decision believing that focusing on the ER classifier, which had the highest accuracy, would reduce the chances of mis-interpreting the visualizations. However, based on our findings, we believe that using these visualization techniques may provide diagnostic information to troubleshoot difficult classification questions.

## Supplementary information

Supplementary Information.
