# Supplementary material for: Deep learned tissue “fingerprints” classify breast cancers by ER/PR/Her2 status from H&E images
Source: Sci Rep. 2020 Apr 29;10:7275. doi: 10.1038/s41598-020-64156-4 (PMC7190637; doi:10.1038/s41598-020-64156-4)
Supplement: Supplementary file 1 — Supplementary Information. [file 41598_2020_64156_MOESM1_ESM.pdf]

Supplementary Materials for

Deep learned tissue "fingerprints" classify breast cancers by ER/PR/Her2 status from H&E images

Author names: Rishi R. Rawat[1], Itzel Ortega[1], Preeyam Roy[1], Fei Sha[2], Darryl Shibata[3], Daniel Ruderman[1], David B. Agus[1]

1 Lawrence J. Ellison Institute for Transformative Medicine, University of Southern California, 2250 Alcazar Street, CSC 240, Los Angeles, CA 90089-9075

2 DASH Center at USC, 1002 Childs Way, MCB 114, Los Angeles, CA 90089-0005

3 Department of Pathology, University of Southern California Health Sciences Campus, NOR 1441 Eastlake Ave., Los Angeles, 90033

Table of contents

Supplemental Table 1: Clinical characteristics of breast cancer patients 2

Supplemental method: Training the fingerprint-based classifier to predict molecular markers 3

Supplemental Figure 1: Embedding of the TMA cores - before color normalization 4

Supplemental Figure 2: TMA core embedding - after color normalization 5

Supplemental Figure 3: Detail of patches from Figure 7 6

Supplemental Table 1: Clinical characteristics of breast cancer patients

Breast Cancer Cohort	TCGA	ABCTB
Number of Patients	939	2531
ER-positive	723	2007
ER-negative	216	524
PR-positive	623	1798
PR-negative	313	716
Her2-positive	151	371
Her2-negative	508	2116
Age (years)		
min	26	
mean	58.2	
max	90	
Stage		
I	156	
II	539	
III	210	
IV	16	
Histologic Grade		
Grade 1		364
Grade 2		904
Grade 3		1004
Tumor Size		
T1	234	
T2	546	
T3	123	
T4	34	
Node Status		
N0	443	
N1	315	
N2	100	
N3	65	
node negative	443	1143
> 1 node positive	480	1388
Metastasis Status		
M0	785	
M1	18	

Supplemental method: Training the fingerprint-based classifier to predict molecular markers

The classifier for each molecular marker was trained in the following manner:

- From the training set, 120 patches were extracted per patient (as per ‘Methods’), and fingerprints were extracted using the pre-trained fingerprint network.
- Then, a matrix containing these fingerprints was created and loaded into memory. The dimensions of the matrix were (N x 120 x 512), where N corresponds to the number of patients in the training set.
- The tensor was reshaped into a data matrix “Xtrain”, with the following dimensions (N * 120 x 512)
- Additionally a “ground truth” vector (length = N) containing the ground truth data for the training patients was prepared. Each value in the vector signifies positive or negative biomarker status by the value of +1 or -1
- Xtrain was passed to a neural network with the following structure:

  Linear (512, 8)
  ReLU
  Linear (8, 1)
  Tanh
- The output of the network after receiving Xtrain is of size (N*120, 1) and is labeled “Pred_unscaled”
- The matrix, “Pred_unscaled” is reshaped to size: N x 120
- Each row in the matrix is normalized as follows:
  - The absolute value is taken of Pred_unscaled and summed over the horizontal dimention, producing a “weight” vector of length N
  - Each row in Pred_unscaled is divided by the corresponding weight vector, producing a matrix Pred_scaled of shape (N x 120)
    - For each row i (0..N):
      - Pred_scaled[i] = Pred_unscaled[i] / weight[i]
  - The output is a matrix of shape N x 120
- Each row in the matrix Pred_scaled is summed to produce a 1D “prediction” vector for the training dataset of length N
- The loss is calculated by the binary cross entropy between the prediction vector and the ground truth vector
- The weights of the model are updated using back propagation and gradient decent. We used the Pytorch implementation of the Adam optimizer with learning rate of 0.0001
- Training was continued until the loss on the cross-validation set stopped decreasing as per the methods section.
- The motivation for applying the normalization step using the average of the absolute value was to allow the network to pay attention to certain areas and ignore others. The Tanh nonlinearity outputs a score from -1 to 1. Areas predicted to be 0 are not contributory to the weight and hence do not factor into the final score for the slide. However, areas that are strongly positive or negative affect the weighting and influence the final patient prediction.

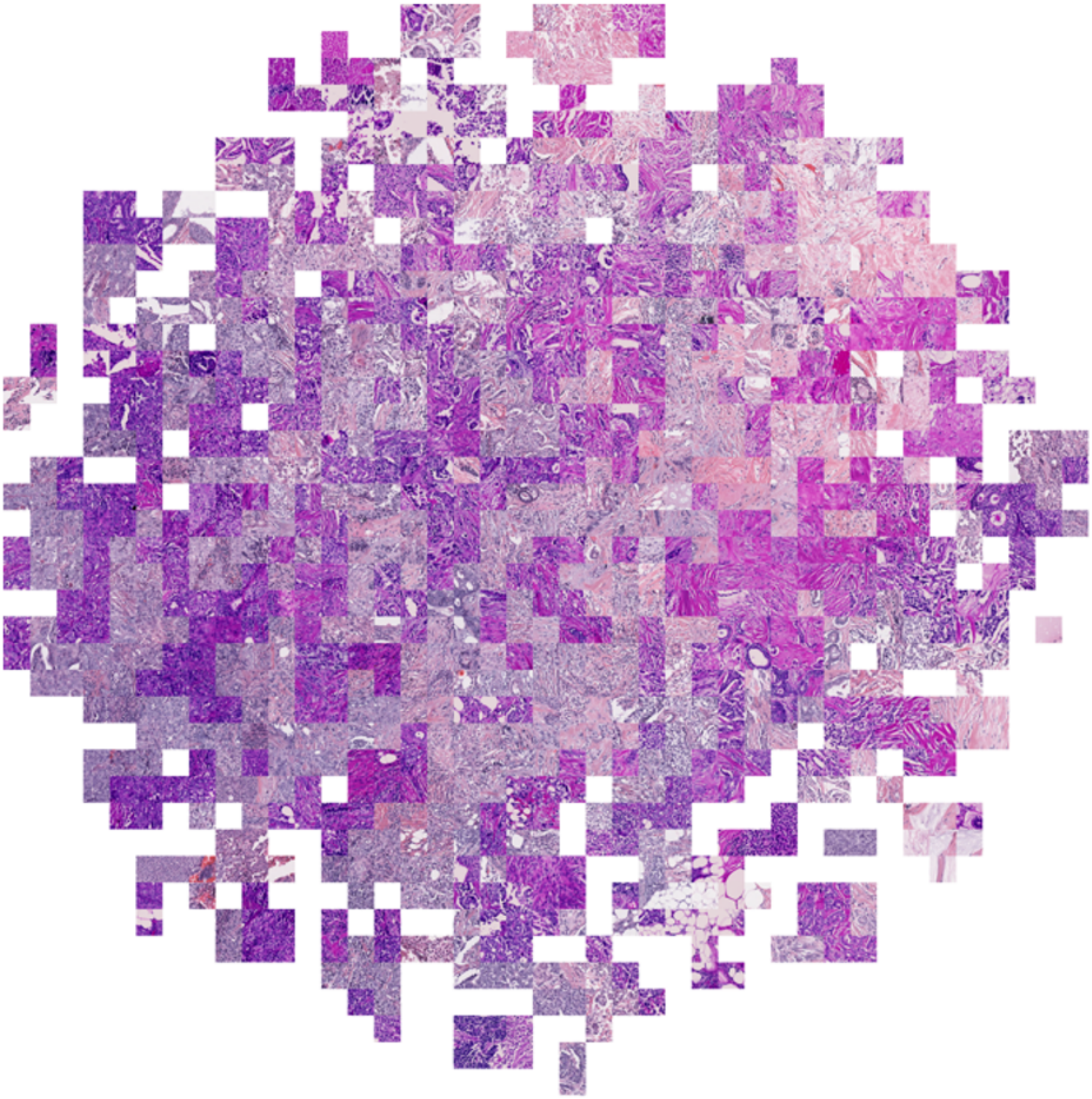

Supplemental Figure 1: Embedding of the TMA cores - before color normalization

Best observed on a screen, rapidly alternating between this image and the next image.

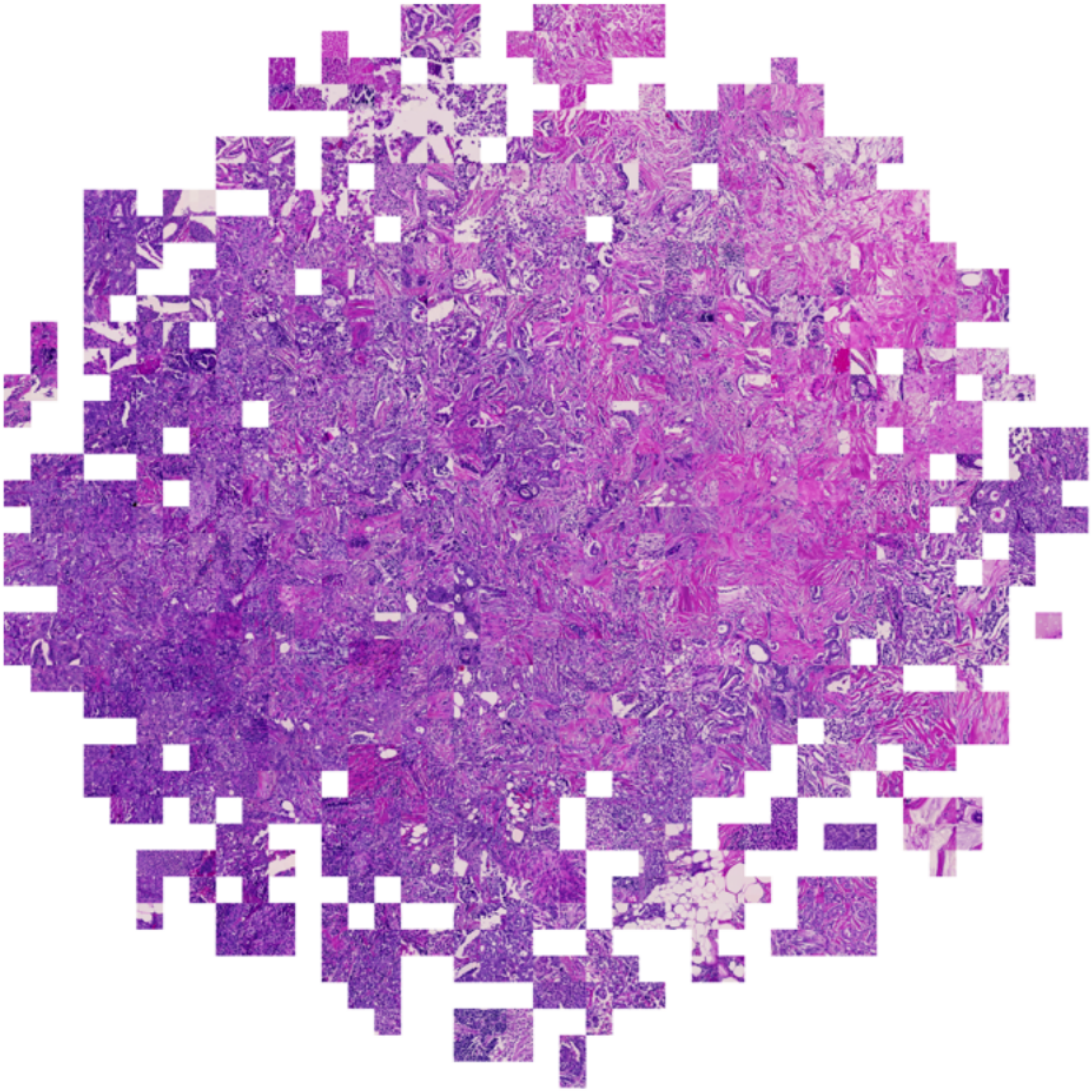

Supplemental Figure 2: TMA core embedding - after color normalization
The Biomax patches have been recolored to look like they were stained at our institution (USC).
Best observed on a screen, rapidly alternating between this image and the next image.

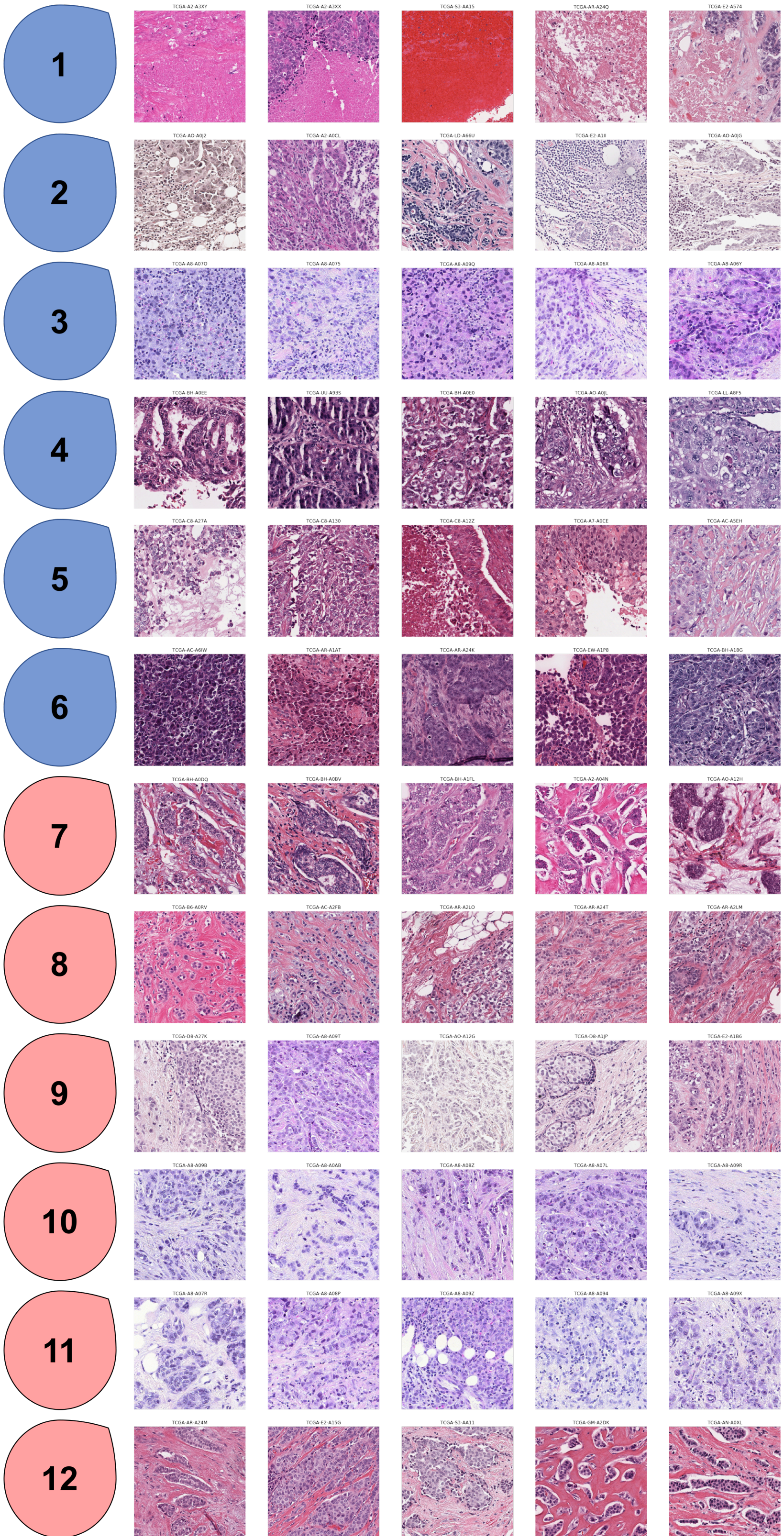

Supplemental Figure 3: Detail of patches from Figure 7

Best viewed on a computer screen
